# The Role of Dendritic Cell Subsets in Recurrent Spontaneous Abortion and the Regulatory Effect of Baicalin on It

**DOI:** 10.1155/2022/9693064

**Published:** 2022-02-17

**Authors:** Nannan Lai, Xiaoxiao Fu, Guozhen Hei, Weiwei Song, Ran Wei, Xiaoxiao Zhu, Qiang Guo, Zhen Zhang, Chu Chu, Ke Xu, Xia Li

**Affiliations:** ^1^Innovative Institute of Chinese Medicine and Pharmacy, Shandong University of Traditional Chinese Medicine, Jinan 250355, China; ^2^Scientific Research Center, The Seventh Affiliated Hospital of Sun Yat-sen University, Shenzhen 518107, China; ^3^Laboratory of Molecular Immunology, School of Basic Medicine, Shandong First Medical University and Shandong Academy of Medical Sciences, Jinan 250062, China; ^4^Shandong Province Maternal and Child Health Care Hospital, Jinan 250013, China; ^5^Department of Obstetrics and Gynecology, The Second Affiliated Hospital of Shandong University of Traditional Chinese Medicine, Jinan 250001, China

## Abstract

Recurrent spontaneous abortion (RSA) is a relevant complication of pregnancy. Aberrant dendritic cell (DC) activities and differentiation have been identified to be involved in RSA, but the underlying mechanisms remain unclear. Baicalin from *Radix Scutellariae* possesses a wide range of pharmacological and biological activities. However, the effect of baicalin on DC function in RSA has not been investigated. Here, we analyzed the changes of peripheral and maternal-fetal interface DC subsets and function in patients and mice with RSA, respectively. Then, we further treated RSA mice with baicalin and analyzed the therapeutic effect and underlying mechanism. We found that DCs from the peripheral blood and decidua of RSA patients and the maternal-fetal of RSA mice were all polarized to conventional DCs, whose proportion was positively correlated with the mice embryo absorption rate. Moreover, DCs from RSA patients and mice showed increased expression of HLA-DR/MHC-II, CD80, and CD86 but decreased expression of CD274 and 33D1. Importantly, baicalin could alleviate embryo resorption of RSA mice by reversing conventional DCs to plasmacytoid DCs and functional molecule expression via inhibiting the STAT5-ID2 pathway. Our research further proved that DCs play an important role in the pathogenesis of RSA and baicalin might be used for treating RSA.

## 1. Introduction

Two or more consecutive spontaneous pregnancy losses before the twentieth week of gestation and excludes ectopic, molar, and biochemical pregnancies are diagnosed as recurrent spontaneous abortion (RSA). The incidence of RSA worldwide is 15-25% of all clinical pregnancies, and the actual pregnancy loss may be closer to 57% if preclinical losses are included [1, 2]. Fetal chromosomal abnormalities, advanced maternal age, alcohol consumption, smoking, and cocaine use are common risk factors of RSA [2, 3]. In addition, chronic diseases, such as autoimmune conditions, diabetes, and celiac disease, as well as infections, such as cervicitis, vaginitis, HIV infection, syphilis, and malaria, are also known pathogenic factors. However, the pathogenesis of more than half RSA patients remains unknown.

Normal pregnancy (NP) is like a successful semiallotransplantation, and its establishment and maintenance depend on the formation of maternal-fetal immune tolerance, in which a variety of immune cells are involved, especially T cells. Activated maternal CD4^+^ T cells can differentiate into different subsets, including Th1, Th2, Th17, and Treg based on the surface phenotype and release cytokines to initiate different maternal-fetal immune responses. Studies showed that the expansion of Th2 and Treg subsets is a sign of successful pregnancy as they play a pivotal role in the establishment and maintenance of maternal tolerance to fetal alloantigen [4, 5]. Correspondingly, the polarization of Th1 and Th17 subsets is closely related to pregnancy failure in RSA patients [6, 7]. Our previous study has confirmed that pregnancy outcomes are closely related to the Th1/Th2/Th17/Treg paradigm, and the paradigm migrates to Th1 and Th17 in RSA mice [8]. However, the initiating factors leading to T cell subsets imbalance during abortion are still unclear.

Dendritic cells (DCs), as the most powerful antigen-presenting cells, play a key role in inducing naïve T cell activation and differentiation into Th1, Th2, Th17, and Treg. Among all DC subsets, conventional DCs (cDCs) and plasmacytoid DCs (pDCs) are mainly focused in pregnancy, as they were found present in the peripheral blood and decidua throughout the pregnancy [9]. So far, there were a limited number of studies focused on the proportion changes of cDCs and pDCs in RSA. Moreover, the findings of cDC and pDC changes described in different studies are diverse as various surface markers or different gestation stages were chosen [10, 11]. Therefore, the role of DC differentiation and functional status in NP and RSA is still poorly understood.

Traditional Chinese medicine has been widely used in the treatment of RSA in China for thousands of years for its good curative effect and few side effects [12, 13]. *Radix Scutellariae* (Huang Qin), a species of flowering plant in the Lamiaceae family, is a commonly used antiabortifacient herb, and baicalin is the predominant flavonoid and bioactive compound of Huang Qin. Studies have confirmed that baicalin is widely used for the treatment of various diseases, including hepatitis, pneumonia, allergy, diabetes, and cancer [14, 15]. However, the therapeutic effect of baicalin on RSA remains unknown. It has been reported that baicalin promotes Treg differentiation, whereas it suppresses the differentiation of Th17 cells and alleviates inflammation [16, 17]. Baicalin also could impair Th1 polarization via significantly inhibiting the expression of DC surface molecules CD80, CD86, major histocompatibility complex (MHC) class I, and MHC class II as well as the levels of IL-12 production [18]. However, the effects of baicalin on DC immune response, especially DC-mediated pregnancy immunity, are still unclear. With all the above in mind, we analyzed the changes of peripheral or maternal-fetal interface DC subsets and function in patients and mice with RSA, respectively. We also treated RSA mice with baicalin and further analyzed the therapeutic effect and mechanism of baicalin on RSA.

## 2. Materials and Methods

### 2.1. Patients

All study participants were patients of the outpatient department of the Department of Obstetrics and Gynecology at the First Affiliated Hospital of Shandong First Medical University between April 2019 and August 2021. All peripheral blood samples and decidual samples were obtained after obtaining informed consent, and all the experiments were approved by the ethics committee of Shandong First Medical University (approved number: H223). Forty-five RSA patients (median age 26 years, range 19-36 years) and 36 NP women (median age 26 years, range 20-36 years) were included in this study (Supplemental Figure 1a). The NP women had no history of miscarriage. The RSA group was defined as women who had experienced two or more consecutive abortions ranging from 6 to 10 weeks of gestation. The diagnosis of RSA was made after excluding any verifiable causes, such as abnormalities of the uterus or cervix, chromosomal abnormalities, infection, endocrine and metabolic diseases, congenital thrombophilia, and autoimmune diseases. More detailed information about the study participants is summarized in Table 1.

### 2.2. Specimen Collection

The human study design was presented in Supplemental Figure 1a. For analysis of the peripheral blood, 3 ml of venous blood was collected from each study participant into ethylene diamine tetra acetic acid blood collection tubes (Becton Dickinson). Samples were centrifuged to collect the upper serum layer, which was snap frozen and stored at -80°C until use. After that, the remaining sample was processed to isolate peripheral blood mononuclear cells (PBMCs) by the Ficoll density-gradient centrifugation. PBMCs were used for RT-PCR and flow cytometry analyses. Decidual samples obtained from induced abortion cases were separated carefully from villi under a stereomicroscope and immersed in normal saline immediately. Then the decidual mononuclear cells were isolated by the Ficoll density-gradient centrifugation after homogenization and filtration through a 40 *μ*M nylon mesh. The decidual mononuclear cells were used for flow cytometry analyses.

### 2.3. Reagents

Baicalin with a purity of 99% according to high-performance liquid chromatography provided in the certificate of analysis was purchased from the National Institute for the Control of Pharmaceutical and Biological Products (Cas: 21967-41-9, Lot: 110715-201318, Beijing, China). Baicalin was dissolved in normal saline in three doses (25 mg/kg/day, 50 mg/kg/day, and 100 mg/kg/day) just before the treatment.

### 2.4. Generation of RSA Mice and Treatment

Eight-week-old CBA/J female (weight 19-21 g), DBA/2 male (weight 20-22 g), and BALB/c male (weight 20-22 g) mice were purchased from Beijing Biotechnology Co., Ltd. All animal experiments were performed in accordance with the guidelines for the Care and Utilization of Laboratory Animals (Shandong First Medical University) and were approved by the Institutional Animal Care and Use Committee of Shandong First Medical University (approved number: M452). Mice were housed on a switching 12-hour light-dark cycle at ambient temperature (25 ± 2°C) with relative humidity (50 ± 10%) in independently ventilated pathogen free cages with corn cobs as bed changed every other day. All mice had free access to standard rodent chow and water. Newly purchased mice were housed for 1 week to acclimate; then all animals were randomly assigned to groups according to study design.

The mice study design was presented in Supplemental Figure 1b and 1c. CBA/J females were naturally mated to BALB/c males to establish the NP model (CBA/J×BALB/c) and to DBA/2 males to establish an RSA (CBA/J×DBA/2) model [19]. Neither females were hormone-induced, nor were males experienced sex before mating. Mated mice were inspected every morning for vaginal plugs. The day when a plug became visible was designated as day 0.5 of pregnancy [20], and mice were euthanized on day 12.5 based on the pregnancy feature of RSA mice [21].

Before baicalin treatment, mice were mated and divided into three groups: RSA group (*n* = 15), NP group (*n* = 15), and unmated CBA/J female mice as the unpregnant group (*n* = 15) (Supplemental Figure 1c). For analysis of baicalin treatment, newly purchased mice were mated. The NP group (*n* = 10) was kept as a positive control group, and RSA mice were randomly divided into four groups: RSA with normal saline group, RSA with baicalin low-dose group (25 mg/kg/day) (RSA + baicalin low-dose, *n* = 10), RSA with baicalin middle-dose group (50 mg/kg/day) (RSA + baicalin middle-dose, *n* = 10), and RSA with baicalin high-dose group (100 mg/kg/day) (RSA + baicalin high-dose, *n* = 10) (Supplemental Figure 1c). The appropriate dosage for baicalin was selected referring to the former research on this compound [22]. Pregnant mice were treated with gavage every day at the corresponding dose in 2002 *μ*l of saline, respectively, from day 0.5 of pregnancy until euthanized.

### 2.5. Fetal Resorption Rate Analysis

The uterus was collected and taken pictures. The number of resorbed embryos and viable embryos were calculated by embryo absorptivity (*R*) = Re/(Re + *F*) as previously described [23], where Re is the number of resorbed embryos and *F* is the number of viable embryos.

### 2.6. Mice Maternal-Fetal Interface Mononuclear Cell Preparation

The maternal-fetal units were harvested and removed from the uterine implantation sites on day 12.5. After removal of the placenta, the decidual tissues were cut into pieces and then lightly ground on the 200-mesh net, washed with 1×PBS containing 0.25% trypsin (Sigma, St. Louis, USA) and 50 U DNase I (Sigma, USA). The cell suspensions were laid carefully on Lymphocyte Separation Media (MD Pacific, Tianjin, China) and then centrifuged at 2000 rpm for 25 min. The middle cellular fraction was recovered and washed twice in 1×PBS at 1500 rpm for 7 min. Cells were used for flow cytometry, qRT-PCR, and western blot analysis.

### 2.7. Antibodies and Flow Cytometry

The following antibodies were used: anti-human CD11c-FITC (clone 3.9), anti-human CD86-PE (clone IT2.2), anti-human CD80-PE-Cy5 (clone 2D10.4), anti-human HLA-DR-PE-Cy5/Percp-Cy5.5 (clone LN3), anti-human CD123-PE (clone 6H6), anti-human CD274-PE (clone MIH1), anti-mouse CD11c-FITC (clone N418), anti-mouse MHC-II-PC5 (clone M5/114.15.2), anti-mouse 33D1-PE (clone 33D1), anti-mouse B220-PE (clone RA3-6B2), and anti-mouse CD274-PE (clone MIH5) were purchased from Invitrogen; anti-mouse CD86-PC5 (clone GL1) and anti-mouse CD80-PE (clone 16-10A1) were purchased from BD Biosciences. Prepared PBMCs or mice maternal-fetal interface mononuclear cells were washed with cold PBS containing 2% FBS and incubated with diluted antibodies at 4°C for 15 min. The cells were then washed twice with cold PBS containing 2% FBS, resuspended in 0.3 ml of PBS containing 2% FBS, and analyzed with a FACSVerse flow cytometer using the FACSuite software.

### 2.8. Quantitative Real-Time PCR (qRT-PCR)

Total RNA was extracted using TRIzol Reagent (CWBIO, Beijing, China) following the manufacturer's instruction. RNA was reverse transcribed with the PrimeScript 1st strand cDNA synthesis kit (Takara, Dalian, China) according to the manufacturer's instructions. Human cDNA was amplified by PCR with 2×PCR reagent (TIANGEN, Beijing, China) using HLA-DR, CD80, CD86, CD274, and *β*-actin primers, respectively. Amplification was conducted at 94°C for 3 min followed by 32 cycles of 94°C for 30 s, 58°C for 30 s, and 72°C for 1 min, and an extra extension at 72°C for 5 min for a total of 20 *μ*l. Samples were run on a 1.5% agarose gel and visualized by staining with ethidium bromide. The Alpha Imager 2200 software (Alpha Innotech Corporation, Santa Clara, CA, USA) was used for quantitative analysis. Mouse MHC-II, CD80, CD86, CD274, 33D1, STAT5, E2-2, and ID2 expressions were analyzed by qPCR using 2×SYBR Green (Invitrogen, Waltham, MA, USA) performed on the Applied Biosystems 7500 instrument (Applied Biosystems, Foster, USA). The PCR was implemented according to the following parameters: 95°C for 3 min, 40 cycles at 95°C for 15 s, 60°C for 30 s, 72°C for 30 s, 60°C for 60 s, and 95°C for 15 s in a total of 20 *μ*l. For each sample, the amplification reaction was performed in triplicates. Relative RNA quantification was performed via the comparative 2^−*ΔΔ*Ct^ method. The relative expression level of genes was normalized to the level of *β*-actin expression in each sample. The primer sequences are shown in Table 2.

### 2.9. ELISA

The serum supernatants were collected and stored at −80°C until use. Human IL-6, IL-12, IL-10, and TGF-*β* cytokine production was analyzed by ELISA kits (all from Thermo Fisher Scientific, USA, Cat: 88-7066-22; 88-7126-22; 88-7106-22; and BMS249-4) according to the manufacturer's instructions. Absorbance was detected using a microplate reader (Thermo Fisher, USA) at 450 nm. All samples were assayed in triplicates.

### 2.10. Western Blot

Cells were lysed in the RIPA buffer (Thermo Scientific, Massachusetts, USA) with protease and phosphatase inhibitor (Thermo Scientific, Massachusetts, USA) on ice for 30 min and then centrifuged at 12000 rpm for 15 min at 4°C. Protein concentration was determined by the BCA protein assays kit (Thermo Scientific, Massachusetts, USA) according to the manufacturer's instruction. Then, protein supernatants were separated by SDS-PAGE and transferred onto a polyvinylidene difluoride (PVDF) membrane in the transfer buffer. The membrane was first blocked in TBS containing 0.5% Tween-20 (TBST) and 5% nonfat milk at 37°C for one hour and then incubated in TBST containing phosphorylated STAT5 (pSTAT5) (#4322, Cell Signalling Technology, Danvers, MA, USA), STAT5 (#94205, Cell Signalling Technology, Danvers, MA, USA), E2-2 (Cat: sc-166699, Santa Cruz Biotechnology), ID2 (#3431S, Cell Signalling Technology, Danvers, MA, USA), and GAPDH (#5174, Cell Signalling Technology, Danvers, MA, USA) primary antibodies in 1 : 500 dilutions at 4°C overnight. The membrane was then washed with TBST three times (10 min each) and incubated with HRP-conjugated anti-rabbit IgG secondary antibody (Cat: 7074, Cell Signalling Technology, Danvers, MA, USA) or HRP-conjugated anti-mouse IgG secondary antibody (Cat: 58802, Cell Signalling Technology, Danvers, MA, USA) at 1 : 5000 dilution at 37°C for 1 h. After washing three times (10 min each) in TBST, the proteins were detected using the enhanced chemiluminescence detection system and Luminescent Image Analyzer LAS-4000 mini (Fujifilm, Tokyo, Japan). To determine the target protein products, the relative intensity of the target protein band was deduced from the ratio to that of the synchronous positive control GAPDH.

### 2.11. Statistical Analysis

Data obtained from flow cytometry were analyzed with the FlowJo 7.6 Software (Treestar, Woodburn, OR) and exported to Excel spreadsheets. Results are presented as mean ± standard deviation (SD). The data were proved to be normal distribution (*p* > 0.05) assessed with the Shapiro-Wilk test or Kolmogorov-Smirnov test. Then, unpaired Student's *t*-test was used to compare the significance between NP women and RSA patients. The diagnostic value of pDC and cDC proportion in PBMCs for RSA patients was evaluated using the receiver operating characteristic (ROC) curve. Analysis of variance (one-way ANOVA) followed by Tukey's post hoc test was used to compare the significance among more than two groups in mice study. Correlations between the proportion of DC subsets in mice maternal-fetal and embryo resorption were analyzed by Pearson's correlation and regression tests. The unpaired Student's *t*-test, ROC curve, and Pearson's correlation and regression tests were performed using GraphPad Prism 6 (San Diego, California), and the one-way ANOVA followed by Tukey's post hoc test was performed using the SPSS statistics 26.0 software (IBM, NY). A *p* < 0.05 was considered statistically significant.

## 3. Results and Discussion

### 3.1. Change of DC Subsets and Function in Patients with RSA

**Figure 1:**
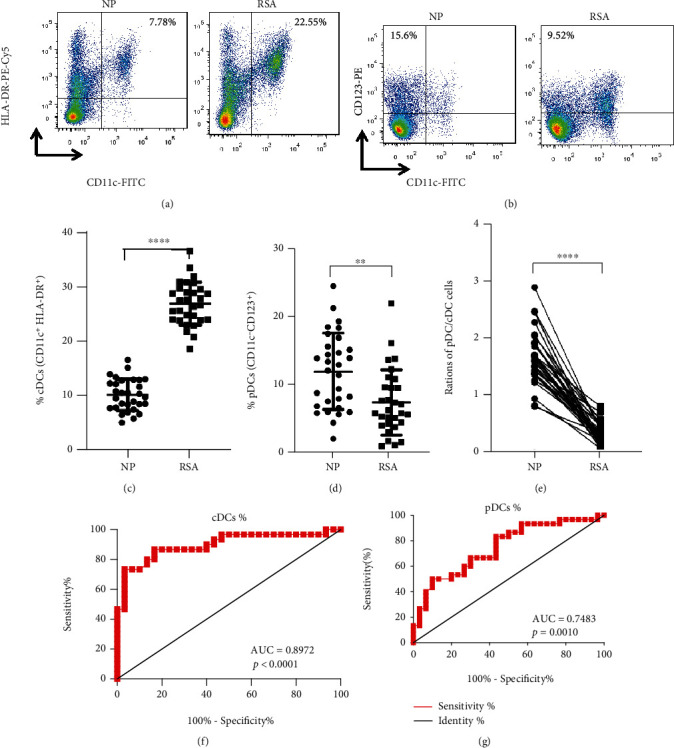
Peripheral DC subsets in patients with RSA. PBMCs were isolated from NP women and RSA patients, analyzed for (a) cDCs (CD11c^+^HLA-DR^+^) and (b) pDCs (CD11c-CD123^+^) by flow cytometry. The proportions of (c) cDCs and (d) pDCs and ratios of (e) pDC/cDC cells in PBMCs from NP women (*n* = 30) and RSA patients (*n* = 30). Data were analyzed by unpaired Student's *t*-test. Mean ± SD are shown. ^∗∗^*p* < 0.01, ^∗∗∗∗^*p* < 0.0001. The diagnostic value of (f) cDCs and (g) pDCs proportion in PBMCs for RSA was assessed by ROC curve (*n* = 30). NP: normal pregnancy; RSA: recurrent spontaneous abortion; AUC: area under the curve.

Considering that the DC subsets present throughout the pregnancy and their percentage were changed in different stages [24], the peripheral cDCs (CD11c^+^HLA-DR^+^) and pDCs (CD11c-CD123^+^) in patients with RSA were detected by flow cytometry. Compared with NP group, the proportion of cDCs was significantly increased in RSA patients (*p* < 0.0001), while the pDC proportion (*p* < 0.01) and pDC/cDC ratio (*p* < 0.0001) were robustly decreased in RSA patients (Figures 1(a)–1(e)). Moreover, ROC analysis revealed that the proportion of cDCs and pDCs in PBMCs could sensitively discriminate RSA with an area under the curve (AUC) of 0.8972 (95% confidence interval: 0.8140-0.9804, *p* < 0.0001) and 0.7483 (95% confidence interval: 0.6247-0.8719, *p* = 0.001), respectively (Figures 1(f) and 1(g)). Importantly, the proportion of cDCs in the decidua of RSA patients was also significantly higher than that in the NP group (*p* < 0.01), while the pDCs proportion (*p* < 0.01) and pDC/cDC ratio (*p* < 0.0001) were significantly reduced (Supplemental Figure 2), which was consistent with the peripheral results.

**Figure 2:**
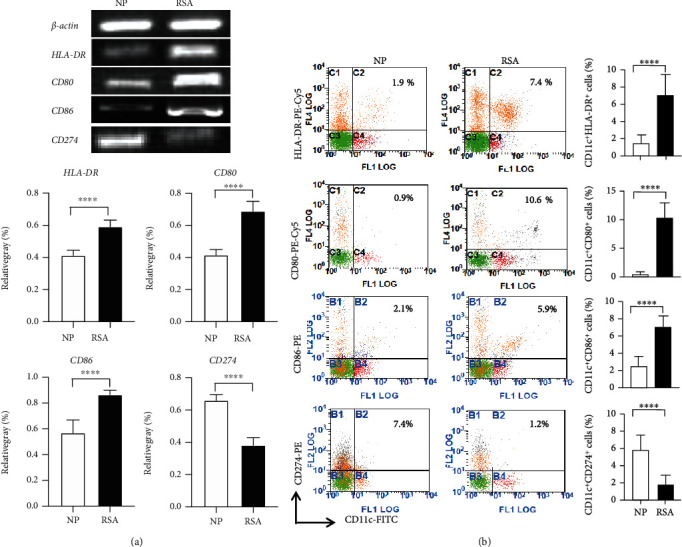
Functional molecule expression of DCs in patients with RSA. (a) HLA-DR, CD80, CD86, and CD274 mRNA expressions in PBMCs of NP women (*n* = 30) and RSA patients (*n* = 30) (upper panel) were detected by RT-PCR. To determine the target mRNA level, the relative intensity of the target DNA band was deduced from the ratio compared to that of a synchronous positive control *β*-actin (lower panel). (b) The expressions of HLA-DR, CD80, CD86, and CD274 in CD11c^+^ cells in PBMCs of NP women (*n* = 30) and RSA patients (*n* = 30) were analyzed by flow cytometry. Data were analyzed by unpaired Student's *t*-test. Mean ± SD are shown. ^∗∗∗∗^*p* < 0.0001. NP: normal pregnancy; RSA: recurrent spontaneous abortion.

**Figure 3:**
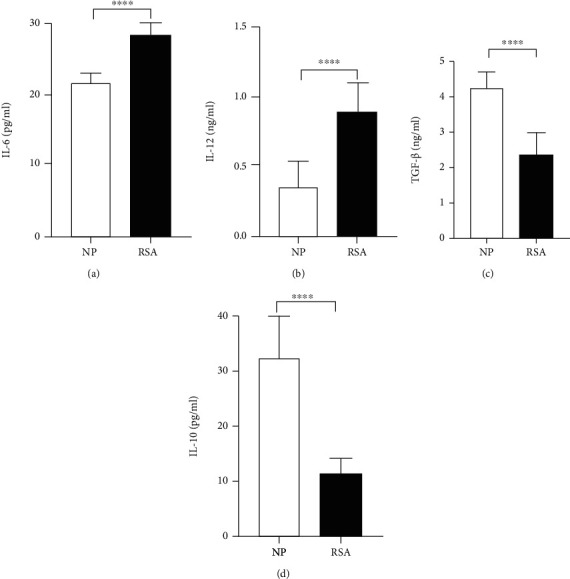
Cytokine expression in peripheral blood of patients with RSA. The concentration of (a) IL-6, (b) IL-12, (c) TGF-*β*, and (d) IL-10 in the serum of NP women (*n* = 22) and RSA patients (*n* = 20) was determined by ELISA. Data were analyzed by unpaired Student's *t*-test. Mean ± SD are shown. ^∗∗∗∗^*p* < 0.0001. NP: normal pregnancy; RSA: recurrent spontaneous abortion.

To analyze whether the DC function of RSA patients has changed, the expression of DC functional molecules and the production of DC-related cytokines in the peripheral were detected. The results showed that the expression of HLA-DR (*p* < 0.0001), CD80 (*p* < 0.0001), and CD86 (*p* < 0.0001) was significantly increased but CD274 (*p* < 0.0001) markedly decreased both in mRNA and protein level in RSA patients as compared to the NP group (Figures 2(a) and 2(b)). The secretion of DC-related cytokines IL-6 (*p* < 0.0001) and IL-12 (*p* < 0.0001) in RSA patients' serum were evidently increased (Figures 3(a) and 3(b)), but TGF-*β* (*p* < 0.0001) and IL-10 (*p* < 0.0001) levels were significantly decreased in comparison with the NP group (Figures 3(c) and 3(d)). These results indicated that the change in the pDC and cDC proportion and in the DC function might play important roles in RSA.

### 3.2. Change of Maternal-Fetal Interface DC Subsets and Function in RSA Mice

**Figure 4:**
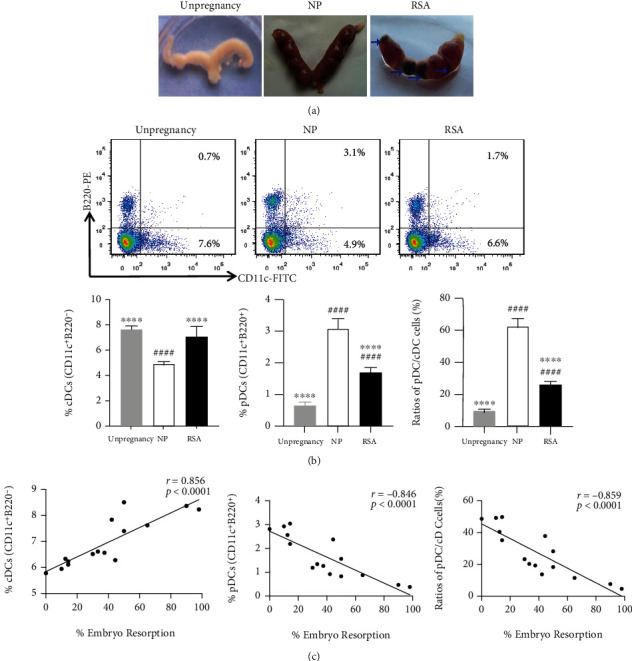
DC subsets in the maternal-fetal interface of RSA mice. CBA/J females were mated to BALB/c males to establish a NP model (CBA/J× BALB/c) and to DBA/2 males to establish an RSA (CBA/J×DBA/2) model. Mice were euthanized on day 12.5 of pregnancy. (a) The representative utera of unpregnant mice (unpregnancy), NP mice, and RSA mice are shown. Blue arrow indicated the absorbed embryo. (b) Mononuclear cells were isolated from maternal-fetal interface of unpregnant mice (unpregnancy, *n* = 15), NP mice (*n* = 15), and RSA mice (*n* = 15), analyzed for cDCs (CD11c^+^B220^−^) and pDCs (CD11c^+^B220^+^) by flow cytometry. Representative FACS profiles (upper). Mean ± SD of the proportions of cDCs and pDCs and ratios of pDC/cDC cells (lower). (c) Correlation between the proportions of cDCs, pDCs, ratios of pDC/cDC cells, and embryo resorption were analyzed. Data were analyzed by Pearson's correlation and regression tests, and one-way ANOVA followed by the Tukey's post hoc test. Mean ± SD are shown. ^∗∗∗∗^*p* < 0.0001, compared with NP; ^####^*p* < 0.0001, compared with unpregnancy. NP: normal pregnancy; RSA: recurrent spontaneous abortion.

To further explore the role of DCs in RSA, CBA/J females were mated to DBA/2 males to establish RSA model mice. The result showed that the uterus of RSA mice presented with visible reabsorbed embryo (blue arrow) because of ischemia, hemorrhage, and necrosis, which was significantly different from the normal embryo of NP mice (CBA/J×BALB/c) and the bihorn uterus of unpregnant mice (Figure 4(a)). Further, the change of pDC and cDC proportion and DC function in maternal-fetal interface was analyzed by flow cytometry (Figure 4(b)). Consistent with our findings in human DCs, the proportion of cDCs (CD11c^+^B220^−^) (*p* < 0.0001) was significantly increased in RSA mice, while the proportion of pDCs (CD11c^+^B220^+^) (*p* < 0.0001) and pDC/cDC ratio (*p* < 0.0001) were significantly decreased in RSA mice as compared to the NP group (Figure 4(b)). In addition, the proportion of pDCs and pDC/cDC ratio in RSA mice were higher than that in the unpregnancy group (*p* < 0.0001), but the cDC proportion between the two groups was comparable (*p* > 0.05). Intriguingly, the proportion of cDCs was positively related to embryo resorption (*r* = 0.856, *p* < 0.0001), whereas both the proportion of pDCs (*r* = −0.846, *p* < 0.0001) and the ratio of pDC/cDC cells (*r* = −0.859, *p* < 0.0001) were negatively related to embryo resorption (Figure 4(c)).

**Figure 5:**
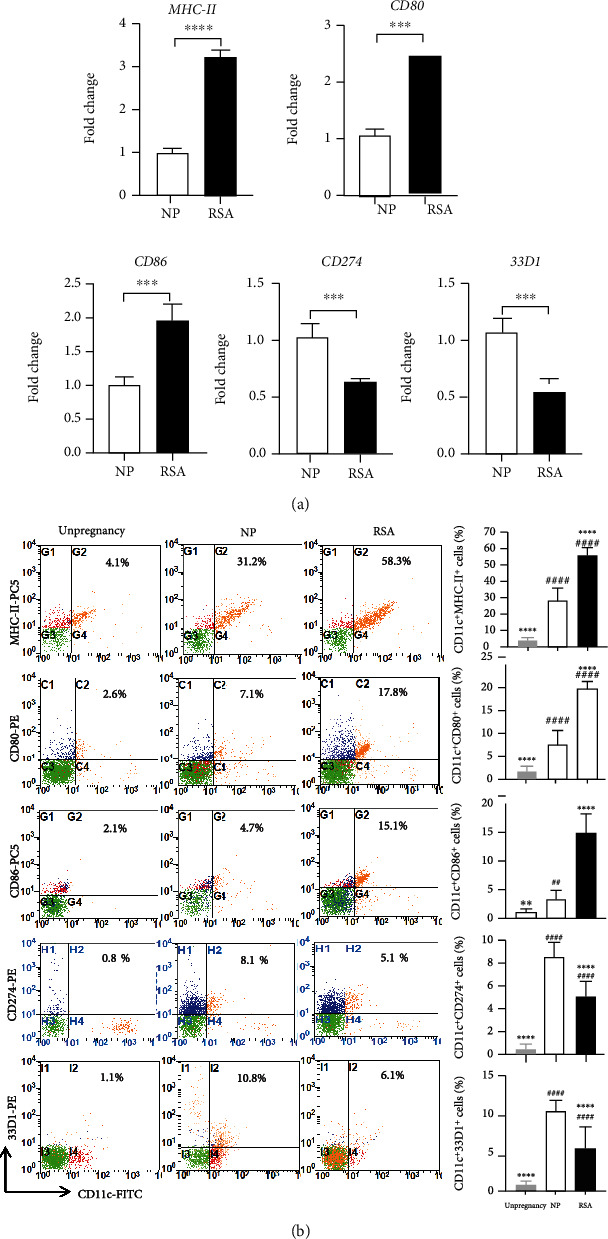
Functional molecule expression of DCs in maternal-fetal interface of RSA mice. (a) The mRNA expression of *MHC-II*, *CD80*, *CD86*, *CD274*, and *33D1* in maternal-fetal interface mononuclear cells of NP mice (*n* = 5) and RSA mice (*n* = 5). (b) The expressions of MHC-II, CD80, CD86, CD274, and 33D1 in CD11c^+^ cells in maternal-fetal interface mononuclear cells of unpregnant mice (*n* = 10), NP mice (*n* = 10), and RSA mice (*n* = 10) were analyzed by flow cytometry. Data were analyzed by unpaired Student's *t*-test and one-way ANOVA followed by the Tukey's post hoc test. Mean ± SD are shown. ^∗∗^*p* < 0.01; ^∗∗∗^*p* < 0.001; ^∗∗∗∗^*p* < 0.0001, compared with NP; ^##^*p* < 0.01; ^####^*p* < 0.0001, compared with unpregnancy. NP: normal pregnancy; RSA: recurrent spontaneous abortion.

Similarly, the expression of DC functional molecules, including MHC-II, CD80, CD86, CD274, and 33D1 was analyzed via qRT-PCR and flow cytometry. As shown in Figure 5, both in mRNA and protein levels, RSA mice exhibited significantly increased upregulation of MHC-II (mRNA: *p* < 0.0001; protein: *p* < 0.0001), CD80 (mRNA: *p* < 0.001; protein: *p* < 0.0001), and CD86 (mRNA: *p* < 0.001; protein: *p* < 0.0001) but reduced expression of CD274 (mRNA: *p* < 0.001; protein: *p* < 0.0001) and 33D1 (mRNA: *p* < 0.001; protein: *p* < 0.0001) compared with the NP group. These results further prove that the change of maternal-fetal interface DC subsets and function is closely related to RSA.

### 3.3. Baicalin Relieves Embryo Resorption of RSA Mice

**Figure 6:**
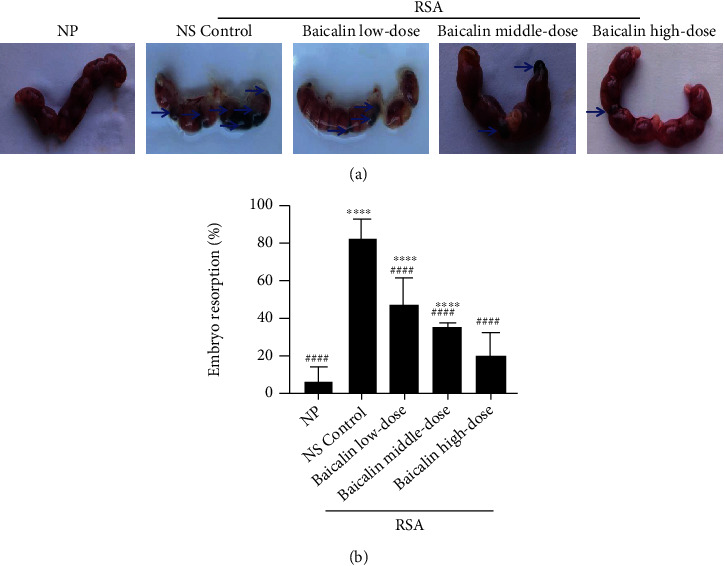
Baicalin alleviates embryo resorption of RSA mice in vivo. (a) Representative utera of NP mice and RSA mice treated with NS control, low-dose baicalin (25 mg/kg/day), middle-dose baicalin (50 mg/kg/day), and high-dose baicalin (100 mg/kg/day) are shown. Arrows indicate the embryo resorption. (b) Embryo resorption rate of groups in (a). Data were analyzed by one-way ANOVA followed by the Tukey post hoc test. Mean ± SD are shown, *n* = 10. ^∗∗∗∗^*p* < 0.0001, compared with NP; ^####^*p* < 0.0001, compared with NS control. NP: normal pregnancy; NS: normal saline; RSA: recurrent spontaneous abortion.

In order to evaluate the potential therapeutic effect of baicalin on RSA mice, randomly grouped RSA mice were treated with normal saline (NS), low-dose baicalin (25 mg/kg/day), middle-dose baicalin (50 mg/kg/day), and high-dose baicalin (100 mg/kg/day) for 12 days, respectively. Compared with the NP group, the NS control group showed severe embryo absorption (blue arrow) with an absorption rate as high as 80% (Figures 6(a) and 6(b)). After baicalin treatment, the proportion of embryo resorption in the baicalin low-dose group was significantly lower than that in the NS control group (*p* < 0.0001, Figure 6(b)), suggesting that it is baicalin rather than normal saline (vehicle) could affect the embryo resorption. Moreover, this phenomenon was more obvious in the baicalin middle- and high-dose groups, and the embryo absorption rate of the high-dose group showed no statistical difference compared with the NP group (*p* > 0.05, Figure 6(b)). Those results indicated that baicalin can effectively alleviate embryo resorption of RSA mice in vivo.

### 3.4. Baicalin Regulates Maternal-Fetal Interface DC Subsets and Function in RSA Mice

**Figure 7:**
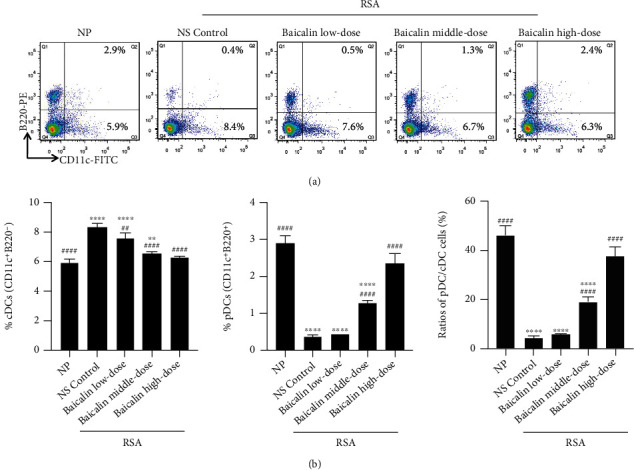
Baicalin regulates maternal-fetal interface DC subsets in RSA mice. (a) Representative FACS profiles of cDCs (CD11c^+^B220^−^) and pDCs (CD11c^+^B220^+^) in maternal-fetal interface mononuclear cells of NP mice and RSA mice treated with NS control, low-dose baicalin (25 mg/kg/day), middle-dose baicalin (50 mg/kg/day), and high-dose baicalin (100 mg/kg/day). (b) The proportions of cDCs and pDCs and ratios of pDC/cDC cells of groups in (a). Data were analyzed by the one-way ANOVA followed by the Tukey post hoc test. Mean ± SD are shown, *n* = 5. ^∗∗^*p* < 0.01; ^∗∗∗∗^*p* < 0.0001, compared with NP; ^##^*p* < 0.01; ^####^*p* < 0.0001, compared with NS control. NP: normal pregnancy; NS: normal saline; RSA: recurrent spontaneous abortion.

To determine whether baicalin has a regulatory effect on DC subsets and function, flow cytometry assay was used to detect the change of pDCs and cDCs and the functional molecule expression at the maternal-fetal interface of RSA mice after baicalin treatment. It was found that the proportion of cDCs in NS control group was significantly increased (*p* < 0.0001), while the proportion of pDCs (*p* < 0.0001) and pDC/cDC ratio (*p* < 0.0001) were markedly decreased in comparison with NP group (Figures 7(a) and 7(b)), which further confirmed the above results. Interestingly, after baicalin treatment, the proportion of cDCs in the baicalin high-dose group was significantly decreased as compared to the NS control group (*p* < 0.0001) and was comparable to NP group (*p* > 0.05). Correspondingly, the proportion of pDCs and pDC/cDC ratio in baicalin middle- or high-dose groups were significantly increased compared with the NS control group (*p* < 0.0001). Moreover, the regulating effect of baicalin on cDCs and pDCs in RSA mice was in a dose-dependent manner (Figure 7).

**Figure 8:**
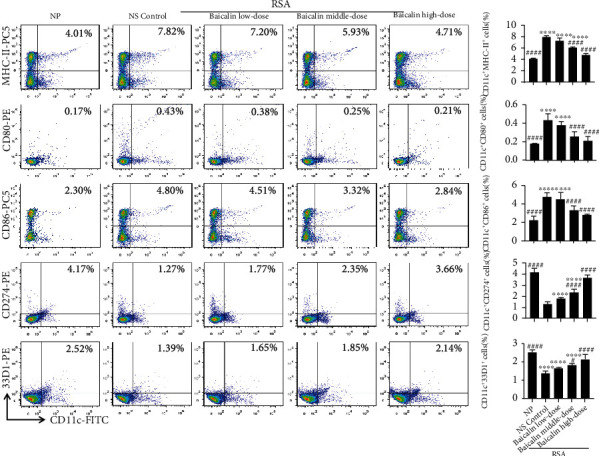
Baicalin regulates maternal-fetal interface DC function in RSA mice. The expressions of MHC-II, CD80, CD86, CD274, and 33D1 in CD11c^+^ cells in maternal-fetal interface mononuclear cells of NP mice and RSA mice treated with NS control, low-dose baicalin (25 mg/kg/day), middle-dose baicalin (50 mg/kg/day), and high-dose baicalin (100 mg/kg/day) were analyzed by flow cytometry. Data were analyzed by one-way ANOVA followed by the Tukey post hoc test. Mean ± SD are shown, *n* = 5. ^∗∗∗∗^*p* < 0.0001, compared with NP; ^#^*p* < 0.05; ^####^*p* < 0.0001, compared with NS control. NP: normal pregnancy; NS: normal saline; RSA: recurrent spontaneous abortion.

Consistent with above results, the NS control group exhibited significantly increased upregulation of MHC-II (*p* < 0.0001), CD80 (*p* < 0.0001), and CD86 (*p* < 0.0001) but reduced expression of CD274 (*p* < 0.0001) and 33D1 (*p* < 0.0001) compared with the NP group (Figure 8). After baicalin treatment, compared with NS control group, the expression of MHC-II, CD80, and CD86 in the baicalin high-dose group was reduced (*p* < 0.0001), and the expression of CD274 and 33D1 was increased (*p* < 0.0001, Figure 8). Moreover, the expression changes of all those functional molecules were in a dose-dependent manner, and the level of baicalin high-dose group was almost to the NP group (*p* > 0.05, Figure 8). These findings suggested that baicalin could prevent RSA by reversing maternal-fetal interface DC subsets and DC-related functional molecule expression in RSA mice.

### 3.5. Baicalin Affects DC Subsets and Function via the STAT5 Pathway to Treat RSA

**Figure 9:**
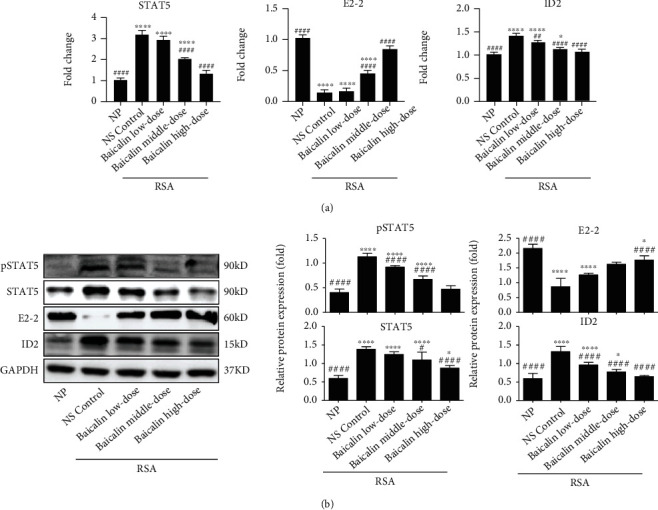
Regulatory role of Baicalin on the STAT5-ID2 signal pathway in RSA mice. (a, b) The mRNA and protein expressions of STAT5, E2-2, and ID2 were analyzed in maternal-fetal interface mononuclear cells of NP mice and RSA mice treated with NS control, low-dose baicalin (25 mg/kg/day), middle-dose baicalin (50 mg/kg/day), and high-dose baicalin (100 mg/kg/day). Data were analyzed by one-way ANOVA followed by the Tukey post hoc test. Mean ± SD are shown, *n* = 3 − 5. ^∗^*p* < 0.05; ^∗∗^*p* < 0.01; ^∗∗∗∗^*p* < 0.0001, compared with NP; ^#^*p* < 0.05; ^##^*p* < 0.01; ^####^*p* < 0.0001, compared with NS control. NP: normal pregnancy; NS: normal saline; RSA: recurrent spontaneous abortion.

In order to further explore the underlying mechanisms of baicalin for regulating DC subsets and function, the levels of STAT5 and its downstream transcription factors, E protein transcription factor TCF4 (E2-2) and ID2 in maternal-fetal derived mononuclear cells, were detected by qPCR and western blot before and after baicalin treatment. Compared with the NP group, the mRNA expression of *STAT5* (*p* < 0.0001) and *ID2* (*p* < 0.0001) in the NS control group was significantly increased, while the mRNA expression of *E2-2* (*p* < 0.0001) was significantly decreased (Figure 9(a)). Correspondingly, the protein level of STAT5 (*p* < 0.0001), pSTAT5 (*p* < 0.0001), and ID2 (*p* < 0.0001) in the NS control group was markedly increased and E2-2 (*p* < 0.0001) was significantly decreased compared with the NP group (Figure 9(b)). Interestingly, baicalin could efficiently reduce *STAT5* and *ID2* mRNA expression and increase *E2-2* mRNA expression in RSA mice in a dose-dependent manner (Figure 9(a)). Meanwhile, baicalin inhibited the protein level of STAT5, pSTAT5, and ID2 in RSA mice and enhanced the protein level of E2-2 in RSA mice (Figure 9(b)). Importantly, there was no significant difference between the baicalin high-dose group and the NP group both in the transcription and protein level of molecules mentioned above (*p* > 0.05, Figure 9). These data suggested that baicalin could reverse the change of DC subsets and function by regulating the STAT5-ID2 signal pathway, thus maintaining maternal-fetal immune tolerance and treating RSA.

## 4. Discussion

Previous studies have confirmed that DCs are involved in the formation and maintenance of maternal-fetal immune tolerance, and the aberrant differentiation and/or functions of DCs may result in RSA [25, 26]. Later, few studies have been published on the subsets and functional changes of DCs in the pathogenesis of RSA, but the results are various [27]. Here, we found an increase in the percentage of cDCs and a decrease in the percentage of pDCs and pDC/cDC ratio in the peripheral blood and decidua of RSA patients as well as RSA mice maternal-fetal interface. Meanwhile, DCs from RSA patients' peripheral blood and RSA mice maternal-fetal interface showed increased expression of HLA-DR, CD80, and CD86 but decreased expression of CD274. Importantly, we proved for the first time that baicalin could relieve embryo resorption of RSA mice. Furthermore, we identified that baicalin could reverse maternal-fetal interface pDC and cDC proportion and functional molecule expression mentioned above via regulating the STAT5-ID2 signal pathway in RSA mice.

Studies have shown that pDCs play an essential role in establishing immune tolerance by inducing Treg development and inhibiting effector CD4^+^ T cell responses [28, 29]. Conversely, cDCs can regulate Th17 differentiation by mediating the expression of proinflammatory factors and anti-inflammatory factors. Moreover, previous study has confirmed that pDCs and cDCs in the decidua could regulate the Th1/Th2 balance to maintain normal pregnancy [30]. Therefore, it is demonstrated that pDCs and cDCs were involved in pregnancy by regulating the Th1/Th2/Th17/Treg subsets. Our research showed that DC subsets in maternal-fetal polarized to pDCs in NP mice compared with unpregnant mice (Figure 4(b)), indicating that pDCs induced immune tolerance to maintain normal pregnancy. On the other hand, DC subsets in RSA patients' peripheral blood and decidua as well as RSA mice maternal-fetal interface shifted to cDCs compared with the NP group, which further confirmed that pDC-mediated immune tolerance was broken. Furthermore, the RSA mice embryonic absorption was significantly positively correlated with the proportion of cDCs and negatively correlated with the proportion of pDCs and the ratio of pDC/cDC at maternal-fetal interface. These results suggest that maternal-fetal DC subset shift to cDCs may lead to RSA, and reversing DC subset shift to pDCs may be an effective target for treating RSA.

Under homeostasis, most DCs in the body are in immature state with low expression of MHC-II molecules, costimulatory molecules CD80, CD86, and CD40 and have strong ability to process antigens but weak ability to extract antigens to stimulate T cell activation, which means that those DCs mainly maintain immune tolerance. We found that DCs from both RSA patients and mice expressed increased HLA-DR/MHC-II, CD80, and CD86, indicating that DCs have changed from an immature state that induces immune tolerance to a mature state that induces T cell activation and further immune response, which might promote RSA formation. CD274, as a B7 family member, plays an important role in inducing T cell activation and differentiation. Previous study showed that DCs from a normal pregnancy peripheral expressed a high level of CD274 compared to DCs in unpregnant women [31]. Our study further proved that DCs from RSA patients and RSA mice were characterized by decreased expression of CD274 compared with the NP group, which might contribute to an abnormal T cell activation and differentiation. Depletion of 33D1^+^ DCs during the perinatal period caused substantial fetal loss probably mediated through Th1 upregulation [32]. We also detected that the expression of 33D1 on DCs in RSA patients and RSA mice was reduced. Mature DCs produce and secrete specific cytokines to activate and direct the development and differentiation of specific T cell subsets. Our research showed that serum levels of IL-12 and IL-6 in RSA patients were significantly higher than that in the NP group, while the expression of TGF-*β* and IL-10 was decreased. It has been reported that the change of these cytokines play a key role in the migration of Th1/Th2/Th17/Treg subsets to Th1/Th17 [33]. Thus, the change of these functional molecular expression and cytokines secretion of DCs could break the balance of maternal-fetal immune tolerance state and result in RSA.

The most important finding of this study is that, for the first time, we proved that the baicalin had a protective role against RSA. Based on previous studies [22] and our preliminary experimental results, we treated RSA mice with gavage every day at baicalin low- (25 mg/kg/day), middle- (50 mg/kg/day), and high- (100 mg/kg/day) dose in 200 *μ*l of normal saline, respectively, from day 0.5 of pregnancy until day 12.5. Then the pregnant mice were euthanized on day 12.5 because the embryo resorption rate was relatively high during this period of RSA mice pregnancy [21]. No animals were found dead during the experiment, so we considered the concentration, timing, and frequency of baicalin administration in vivo study under investigation safe. Recent studies have reported that baicalin can establish Th1 polarization by inhibiting DC maturation [18]. We found that baicalin significantly suppressed the expression of surface molecules MHC-II, CD80, and CD86 in maternal-fetal DCs of RSA mice. Moreover, the decreased expression of CD274 and 33D1 was also increased after baicalin treatment. Importantly, the maternal-fetal interface DC subsets (pDC/cDC) of RSA mice shifted towards pDC after baicalin treatment. Our results indicated that baicalin maintained the maternal-fetal immune tolerance by reversing the functional status and pDC/cDC subsets of maternal-fetal DCs in RSA.

Studies have shown that STAT5 is required for DC activation through the upregulation of costimulatory molecules and enhanced chemokine production [34]. In addition, STAT5 regulates the differentiation of DCs by inducing the expansion of cDCs and inhibiting the development of pDCs [35, 36]. Interestingly, we found that the expression of total STAT5 and pSTAT5 was increased in RSA mice with upregulated costimulatory molecules and expanded cDC proportion. After baicalin treatment, the expression of total STAT5 and pSTAT5 in RSA mice was decreased in a dose-dependent manner. Moreover, the expression of E2-2, which promotes pDC development [37], was increased while the expression of ID2, which breaks the E2-2-driven transcriptional program and induces cDC formation [38], was decreased after baicalin treatment in RSA mice. Previous studies also have demonstrated that STAT5 interacts with the ID2 promoter region [39, 40]. Therefore, baicalin might reverse the functional status and pDC/cDC subsets of maternal-fetal DCs in RSA mice by downregulating the STAT5-ID2 signalling pathway and upregulating the E2-2 expression.

Although the roles of DCs were confirmed and the potential pathway was indicated in RSA, the main limitations of this study were as follows: (a) the clinical sample size of decidual tissues was limited; (b) the makers of DC subsets and function were not diversified enough; and (c) the regulatory mechanisms of baicalin should be further deeply explored. In the future study, we will further explore the underlying mechanism of DC polarization and dysfunction in RSA in order to explain the pathogenesis of RSA and offer novel therapeutic targets for the clinic. In addition, we will further demonstrate the key targets of baicalin for regulating DC polarization and function. Moreover, we will observe the regulatory effects of baicalin on other immune cells such as natural killer cells, macrophages, and T cells at the maternal-fetal interface. Significantly, this study elucidated the pathogenesis of RSA from the perspective of DC immune regulation and demonstrated that baicalin might have potential immunotherapeutic effect on RSA by remodeling maternal-fetal immunotolerance. For further application, it is considerable to optimize the tissue selectivity and improve the stability of baicalin by modifying its chemical structure. In addition, it is necessary to try different combinations of baicalin and other agents and observe the synergy between them.

## 5. Conclusions

In summary, our study showed that the DC subsets shifted to cDCs and presented with high expression of MHC-II/HLA-DR, CD80, and CD86 but reduced the expression of CD274 and 33D1 in RSA patients and mice. Our data also proved that baicalin protected from RSA via reversing cDCs to pDCs and the expression of DC-related functional molecular MHC-II/HLA-DR, CD80, CD86, CD274, and 33D1 through the regulation of the STAT5-ID2/E2-2 pathway. These findings uncover novel immunopharmacological functions of baicalin in treating RSA and suggest new implications for manipulating DC function as potential immunotherapeutic applications in RSA.

## Figures and Tables

**Table 1:** Clinical characteristics of NP women and RSA patients.

Subject	RSA (mean ± SD, *n* = 45)	NP (mean ± SD, *n* = 36)	*p*
Age (years)	25.40 ± 3.41	26.31 ± 4.05	0.27
Number of miscarriages	3.13 ± 0.63	0	<0.0001
Gestation age (weeks)	7.31 ± 1.59	7.18 ± 1.53	0.71

Note: data are presented as mean ± SD, *n* = 45 (RSA), *n* = 36 (NP). Statistical analysis was done by unpaired Student's *t*-test. *p* value, compared with NP. NP: normal pregnancy; RSA: recurrent spontaneous abortion.

**Table 2:** The forward and reverse primer sequences (5′-3′) of human and mouse genes used for PCR.

Primers	Sequence (5′-3′)
Human *HLA-DR*-F	AGCTGTGGACAAAGCCAACCTG
Human *HLA-DR*-:	CTCTCAGTTCCACAGGGCTGTT
Human *CD80*-F	AGGGGAAATGTCGCCTCTC
Human *CD80*-R	GTCCGGTTCTTGTACTCGGG
Human *CD86*-F	AGCACAGACACACGGATGAG
Human *CD86*-R	AGGCCGCTTCTTCTTCTTCC
Human *CD274*-F	TTGCTGAACGCCCCATACAA
Human *CD274*-R	GATGAGCCCCTCAGGCATTT
Human *β-actin*-F	AGCGAGCATCCCCCAAAGTT
Human *β-actin*-R	GGGCACGAAGGCTCATC
Mice *MHC-II*-F	CTGTCACGGTCGAGTGGAAA
Mice *MHC-II*-R	CCTGTTGGCTGAAGTCCAGA
Mice *CD80*-F	CCTCAAGTTTCCATGTCCAAGGC
Mice *CD80*-R	GAGGAGAGTTGTAACGGCAAGG
Mice *CD86*-F	ACGTATTGGAAGGAGATTACAGCT
Mice *CD86*-R	TCTGTCAGCGTTACTATCCCGC
Mice *CD274*-F	TGCGGACTACAAGCGAATCACG
Mice *CD274*-R	TGCGGACTACAAGCGAATCACG
Mice *33D1*-F	ACCAGCAACCTGAACACAAGT
Mice *33D1*-R	ATCTTGGTTGGGCTCACCTT
Mice *STAT5*-F	CTCCGCAGCACCAGGTAAA
Mice *STAT5*-R	ACCTCGATGGGGAAATGCTG
Mice *E2-2*-F	AGACACTCGCTCATGGTTGG
Mice *E2-2*-R	TTGGCAGGAGAGAATGGCTG
Mice *ID-2*-F	ACTCGCATCCCACTATCGTC
Mice *ID-2*-R	GATGTCCGTGTTCAGGGTGG
Mice *β-actin*-F	GTGACGTTGACATCCGTAAAGA
Mice *β-actin*-R	GCCGGACTCATCGTACTCC

## Data Availability

The data used to support the findings of this study are available from the corresponding author upon request.

## Supplementary Materials

Supplementary Materials Figure S1: diagram of study design. NP: normal pregnancy; NS: normal saline; RSA: recurrent spontaneous abortion. Figure S2: decidual DC subsets in patients with RSA. cDCs (CD11c^+^HLA-DR^+^) (a) and pDCs (CD11c^−^CD123^+^) (b) in the decidua of RSA patients (*n* = 15) and NP women (*n* = 6) were analyzed by flow cytometry. The proportions of cDCs (c) and pDCs (d) and ratios of pDC/cDC cells (e) in the decidua of NP women (*n* = 6) and RSA patients (*n* = 15). Data were analyzed by unpaired Student's *t*-test. Mean ± SD are shown. ^∗∗^*p* < 0.01; ^∗∗∗∗^*p* < 0.0001. NP: normal pregnancy; RSA: recurrent spontaneous abortion.
