# Supplementary material for: The Role of Dendritic Cell Subsets in Recurrent Spontaneous Abortion and the Regulatory Effect of Baicalin on It
Source: J Immunol Res. 2022 Feb 17;2022:9693064. doi: 10.1155/2022/9693064 (PMC8872676; doi:10.1155/2022/9693064)
Supplement: Supplementary Materials — Figure S1: diagram of study design. NP: normal pregnancy; NS: normal saline; RSA: recurrent spontaneous abortion. Figure S2: decidual DC subsets in patients with RSA. cDCs (CD11c+HLA-DR+) (a) and pDCs (CD11c−CD123+) (b) in the decidua of RSA patients (n = 15) and NP women (n = 6) were analyzed by flow cytometry. The proportions of cDCs (c) and pDCs (d) and ratios of pDC/cDC cells (e) in the decidua of NP women (n = 6) and RSA patients (n = 15). Data were analyzed by unpaired Student's t-test. Mean ± SD are shown. ∗∗p < 0.01; ∗∗∗∗p < 0.0001. NP: normal pregnancy; RSA: recurrent spontaneous abortion. [file 9693064.f1.docx]

# Journal of Immunology Research

# The role of dendritic cell subsets in recurrent spontaneous abortion and the regulatory effect of baicalin on it

Nannan Lai,^2*^ Xiaoxiao Fu,^5,1*^ Guozhen Hei,^3*^ Weiei Song,^4^ Ran Wei,^5^ Xiaoxiao Zhu,^5^ Qiang Guo,^5^ Zhen Zhang,^5^ Chu Chu,^1^ Ke Xu,^5^ and Xia Li^1,5^

^1^ Innovative Institute of Chinese Medicine and Pharmacy, Shandong University of Traditional Chinese Medicine, Jinan 250355, China

^2^ Scientific Research Center, The Seventh Affiliated Hospital of Sun Yat-sen University, Shenzhen 518107, China

^3^ Shandong Province Maternal and Child Health Care Hospital, Jinan 250013, China

^4^ Department of Obstetrics and Gynecology, The Second Affiliated Hospital of Shandong University of Traditional Chinese Medicine, Jinan 250001, China

^5^ Laboratory of Molecular Immunology, School of Basic Medicine, Shandong First Medical University & Shandong Academy of Medical Sciences, Jinan 250062, China

*The first three authors contributed equally to this work.

Correspondence should be addressed to Xia Li; 60230033@sdutcm.edu.cn

## Supplementary Materials

**
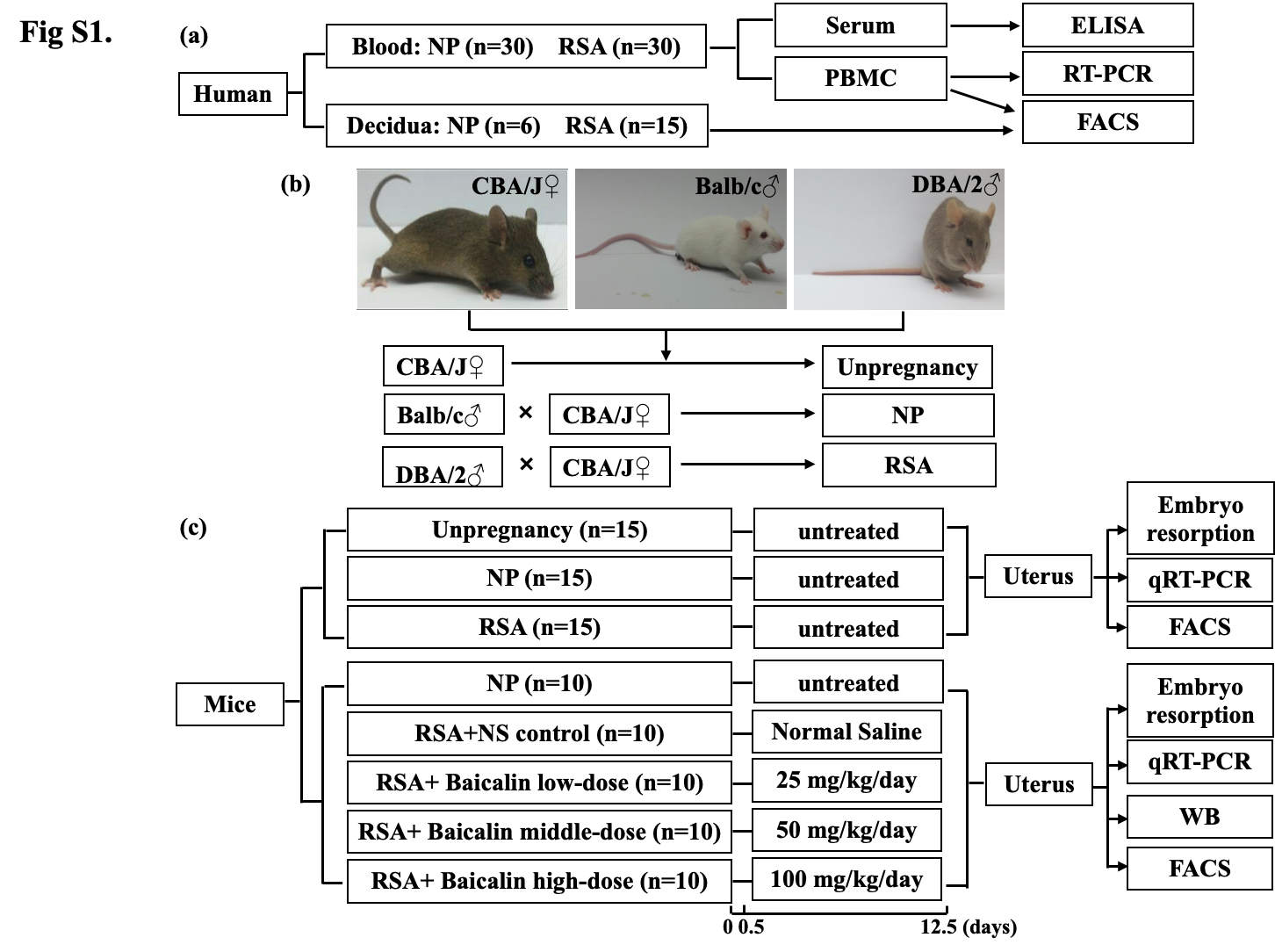
**

**Supplementary Figure 1:** Diagram of study design. NP: normal pregnancy; NS: normal saline; RSA: recurrent spontaneous abortion.


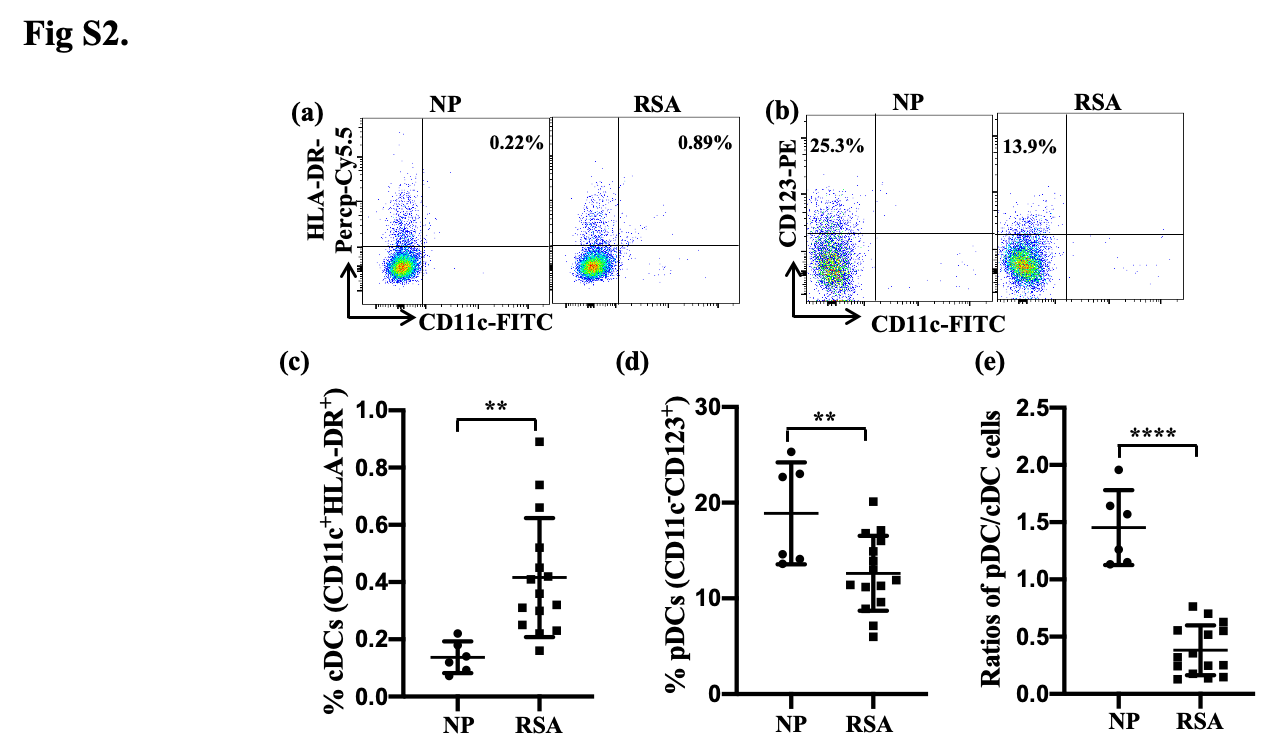


**Supplementary Figure 2:** Decidual DC subsets in patients with RSA. cDCs (CD11c^+^HLA-DR^+^) (a) and pDCs (CD11c^-^CD123^+^) (b) in the decidua of RSA patients (n=15) and NP women (n=6) were analyzed by flow cytometry. The proportions of cDCs (c) and pDCs (d) and ratios of pDC/cDC cells (e) in the decidua of NP women (n=6) and RSA patients (n=15). Data were analyzed by unpaired student’s *t*-test. Mean ± SD are shown. ^**^*p*<0.01; ^****^*p*<0.0001. NP: normal pregnancy; RSA: recurrent spontaneous abortion.
